# Efficacy and Safety of Topical Steroid Therapy for Oral Mucositis During Cancer Chemotherapy: A Scoping Review

**DOI:** 10.7759/cureus.91755

**Published:** 2025-09-07

**Authors:** Madoka Funahara, Sakiko Soutome, Yuki Sakamoto, Teruyuki Niimi

**Affiliations:** 1 School of Oral Health Sciences, Faculty of Dentistry, Kyushu Dental University, Kitakyusyu, JPN; 2 Department of Oral Health, Nagasaki University Graduate School of Biomedical Sciences, Nagasaki, JPN; 3 Oral Surgery, Kansai Medical University Medical Center, Moriguchi, JPN; 4 Division of Research and Treatment for Oral and Maxillofacial Congenital Anomalies, School of Dentistry, Aichi Gakuin University, Nagoya, JPN

**Keywords:** cancer chemotherapy, efficacy, oral mucositis, safety, scoping review, topical corticosteroid

## Abstract

Oral mucositis (OM) is a common and debilitating complication of cancer chemotherapy and targeted therapies, significantly affecting patients’ quality of life and potentially leading to treatment delays or dose reductions. Although topical corticosteroids have demonstrated anti-inflammatory effects, their clinical use for OM remains limited, particularly in countries such as Japan, due to regulatory and safety concerns. This scoping review examined existing evidence on the efficacy and safety of topical corticosteroid therapy-administered via mouthwash or ointment, for the prevention and management of OM in patients undergoing cancer pharmacotherapy. A comprehensive literature search was conducted using PubMed and Scopus to identify English-language studies published up to June 2025, and six eligible studies were included in the qualitative synthesis. The reviewed studies demonstrated that topical corticosteroid therapy, particularly dexamethasone-based mouthwashes and ointments, was associated with significant reductions in OM incidence and severity, with no adverse events such as oral candidiasis reported. Randomized controlled trials by Niikura et al. and Kuba et al. provided the most robust evidence, supporting both efficacy and safety. Moreover, combined use with professional oral care may further enhance therapeutic outcomes. Overall, topical corticosteroids appear to be a safe and effective intervention for managing chemotherapy- and targeted therapy-induced OM. However, the evidence remains limited by small sample sizes and heterogeneous study designs, underscoring the need for large-scale clinical trials to standardize treatment protocols, evaluate long-term safety, and inform future guidelines and policy development.

## Introduction and background

Oral mucositis (OM) is one of the most common and debilitating complications of cancer pharmacotherapy, particularly in patients undergoing high-dose chemotherapy, radiotherapy to the head and neck region, or treatment with molecularly targeted agents such as mammalian target of rapamycin inhibitors (mTOR inhibitors, which block cell growth signaling) and tyrosine kinase inhibitors (agents that inhibit enzymes involved in cancer cell proliferation) [1-4]. It is characterized by erythema, ulceration, and pain of the oral mucosa, leading to nutritional impairment, increased risk of systemic infection, reduced quality of life (QOL), and potentially, treatment interruption or dose reduction [5-7].

The pathophysiology of OM involves a complex cascade of biological events, including direct cytotoxic injury, generation of reactive oxygen species, activation of proinflammatory cytokines (e.g., TNF-α, IL-1β), and subsequent epithelial cell apoptosis and ulceration [8,9]. Given this multifactorial process, management strategies for OM must ideally address both inflammation and microbial superinfection, while being safe and well-tolerated by patients.

A variety of pharmacological and nonpharmacological interventions have been proposed for OM prevention, including benzydamine (a topical anti-inflammatory mouthwash), cryotherapy (oral cooling to reduce mucosal injury during chemotherapy), low-level laser therapy (to promote mucosal healing), and oral care protocols, many of which are recommended in the 2020 Multinational Association of Supportive Care in Cancer/International Society of Oral Oncology (MASCC/ISOO) clinical practice guidelines [2]. However, the role of topical corticosteroids, including steroid-containing mouthwashes and ointments, remains controversial and has not yet been formally endorsed in international guidelines. This controversy reflects concerns about opportunistic infections such as oral candidiasis, the limited number and heterogeneity of randomized controlled trials, and the lack of approved commercial formulations in many countries. Nevertheless, a growing body of evidence supports their anti-inflammatory effects and local efficacy in reducing the severity and incidence of OM in both chemotherapy and targeted therapy settings.

In recent years, evidence has been accumulating that supports a reduction in the severity and incidence of OM. Specifically, the SWISH trial, a phase II study in breast cancer patients receiving everolimus, demonstrated that dexamethasone mouthwash significantly reduced the incidence of OM in patients receiving everolimus [7]. Furthermore, randomized controlled trials by Kuba et al. and Niikura et al. confirmed the efficacy and safety of dexamethasone-based mouthwashes and ointments in broader oncology populations [10,11].

Nevertheless, the clinical adoption of topical corticosteroids for OM remains limited in some countries, including Japan, due to several challenges: (1) the off-label status of steroid mouthwashes, (2) the lack of approved commercial formulations, and (3) persistent concerns about side effects such as oral candidiasis [12-14]. These barriers persist despite clinical data suggesting a low incidence of such adverse effects, particularly when proper oral hygiene is maintained.

Moreover, while international studies have explored a variety of formulations (e.g., dexamethasone, hydrocortisone, betamethasone) and delivery methods (e.g., rinse, gel, ointment), the heterogeneity of these interventions and variation in clinical settings underscore the need for a comprehensive mapping of the existing evidence base.

Therefore, this scoping review aims to systematically examine and summarize the current literature on the use of topical corticosteroid therapy, administered via mouthwash or ointment, for the prevention and management of OM in patients receiving cancer pharmacotherapy. In doing so, we aim to (1) identify the scope and quality of available evidence, (2) clarify gaps in knowledge and clinical practice, and (3) inform future research and policy, particularly in the context of Japanese oncology and supportive care.

## Review

Search strategy and inclusion criteria

A comprehensive search was performed using PubMed and Scopus, focusing on English-language studies published from database inception up to June 2025. Search terms targeted OM, topical steroids, chemotherapy, and related treatment interventions. Studies were included if they investigated the prevention or treatment of OM via topical steroid administration (mouthwash or ointment) during cancer pharmacotherapy (Table 1). 

**Table 1:** Detailes of data sources This table summarizes the key components of the search strategy used to identify relevant studies on the use of topical steroids for oral mucositis during cancer pharmacotherapy. It includes the databases searched, additional sources consulted, the search period, language restrictions, and the main search terms

Search details	
Database	PubMed, Scopus
Other resources	Reference lists and a manual search in key journals
Search period	From database inception until June 2025
Language	Primary studies published in English
Search terms	"cancer" AND "chemotherapy"AND "oral" AND "mucositis" AND "steroid"

Study selection

**Figure 1:**
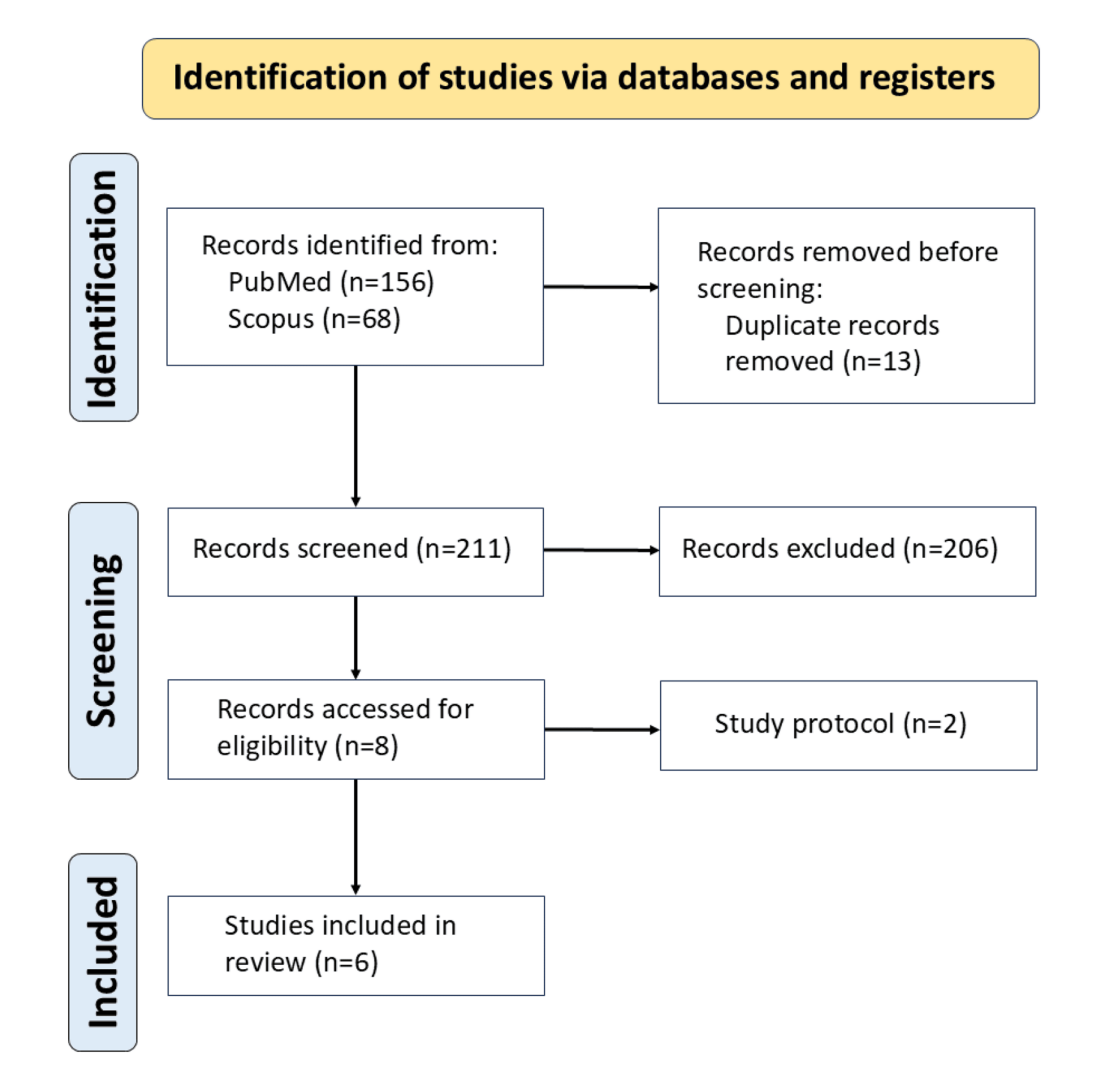
Identification of studies via databases and registers A total of 224 records were identified through database searches (PubMed and Scopus). After removing 13 duplicates, 211 records underwent title and abstract screening. Following full-text assessment of potentially relevant articles, eight studies met the initial inclusion criteria. Two were excluded as protocol papers, resulting in six original studies included in the qualitative synthesis

The search yielded 224 records. After removal of duplicates, 211 records were screened at the title/abstract level. Full texts of potentially eligible articles were assessed, and eight met the prespecified inclusion criteria. Two protocol papers were excluded, leaving six original studies for inclusion in the qualitative synthesis (Figure 1) [7,10,11,15-17]. Four reviewers (FM, SS, YS, and NT) independently conducted the searches and participated in study selection; any discrepancies regarding eligibility were resolved through discussion until consensus was reached. The six included studies were reviewed in detail, and their key findings were collaboratively extracted and summarized by the reviewer team. All six of these studies were conducted at university hospitals and their affiliated cancer centers, and no private practice settings were included.

Summary of included studies

Raeessi et al. [15]

This is a randomized trial comparing honey-based mouthwashes (with betamethasone, coffee, or alone) in 62 patients. All groups showed improvement, with the honey + coffee group demonstrating the greatest benefit.

Rugo et al. (SWISH trial) [7]

This is a phase II trial in 85 breast cancer patients receiving everolimus and exemestane. Dexamethasone mouthwash significantly reduced grade ≥2 mucositis (two cases), compared with historical controls in the BOLERO-2 trial (159 of 482 patients).

Hattori et al. [16]

This is the study of hydrocortisone-containing mouthwash in 29 patients receiving everolimus. Higher mucositis incidence was reported than in the SWISH trial. The authors noted possible bias from historical control comparisons.

Fernández-Sala et al. [17]

Dexamethasone mouthwash was used in 14 patients on various anticancer therapies. Reported as safe and effective, but lacked a control group and had a limited sample size.

Niikura et al. [10]

This is a randomized study of 174 breast cancer patients receiving everolimus and exemestane. The intervention group received dental hygiene plus dexamethasone ointment. Mucositis incidence was significantly reduced without any reported adverse effects.

Kuba et al. [11]

This is an RCT in 117 patients receiving chemotherapy. The group using dexamethasone mouthwash had a significantly lower incidence of OM (38%) versus the control (55%).

These studies are summarized in Table 2.

**Table 2:** Summary of included studies The table provides a detailed overview of the six studies included in the qualitative synthesis. It lists the authors, publication year, study setting, reference information, study design, participant characteristics, details of the topical steroid intervention (mouthwash or ointment), control conditions, and the main findings regarding oral mucositis prevention or treatment

Authors	Year	Setting	Study design	Participants	Intervention	Control	Results
Raeessi et al. [15]	2014	Iran	RCT	62 patients with chemotherapy-induced oral mucositis	Betamethasone + honey (n = 21) coffee + honey (n = 21)	Honey (n = 20)	All 3 groups showed a reduction in the severity of oral mucositis, but the honey and coffee group demonstrated the greatest effectiveness
Rugo et al. [7]	2017	US	Phase 2	85 patients with breast cancer receiving everolimus and exemestane	Dexamethasone-based mouthwash	Historical (482 patients in BOLERO-2)	Grade 2 oral mucositis occurred in 2/83 in the intervention group and 159/482 in BOLERO-2 trial
Hattori et al. [16]	2019	Japan	Phase 2	29 patients receiving everolimus	Mouthwash containing hydrocortisone, itraconazole, tetracycline hydrochloride, and chlorpheniramine	(-)	The prophylactic effect of steroid-containing mouthwash could not be demonstrated
Fernández-Sala et al. [17]	2020	Spain	Phase 2	(1) 9 patients receiving everolimus, and (2) 5 patients with oral mucositis due to anticancer drugs	Dexamethasone-based mouthwash	(-)	Dexamethasone mouthwash was safe and effective in the prevention and reduction of stomatitis
Niikura et al. [10]	2022	Japan	RCT	174 patients with breast cancer receiving everolimus and exemestane	Oral care and dexamethasone ointment	Oral hygiene instruction	Oral care and dexamethasone ointment reduced the incidence and severity of oral mucositis
Kuba et al. [11]	2023	Japan	RCT	117 patients with breast cancer receiving epirubicin + cyclophosphamide or docetaxel + cyclophosphamide	Oral care and a dexamethasone-based mouthwash	Oral care	Stomatitis incidence was 55% and 38% in the control and intervention groups. No severe complications were observed

Findings

Topical steroid therapy, whether via mouthwash or ointment, appears to be a promising approach for both prevention and management of chemotherapy- or targeted therapy-induced OM. Several previous studies, including the SWISH trial and RCTs by Niikura et al. and Kuba et al., have demonstrated the efficacy of topical corticosteroids in reducing OM incidence and severity. Our scoping review confirms and extends these findings by systematically summarizing all available evidence across different formulations and cancer therapies. Importantly, no adverse events such as oral candidiasis were reported in any of the six studies reviewed. Combining topical steroids with professional oral care may further enhance prophylactic efficacy.

Discussion

OM continues to present a significant challenge in oncology, particularly in patients receiving chemotherapy or molecularly targeted therapies. This scoping review highlights emerging evidence that supports the use of topical corticosteroids, administered via mouthwash or ointment, as an effective and safe approach for the prevention and management of OM. OM remains a significant barrier to maintaining uninterrupted cancer treatment. Its pathogenesis involves cytokine-mediated inflammation, oxidative stress, and secondary bacterial infection [3,18]. Therefore, prevention strategies ideally combine anti-inflammatory and antimicrobial approaches.

Although the MASCC/ISOO 2020 guidelines include various interventions such as benzydamine, cryotherapy, and laser therapy, they do not recommend topical steroids, likely due to limited high-quality evidence and concerns about candidiasis [19]. Concerns may stem from experiences with inhaled corticosteroids, which are associated with increased oral candidiasis risk in asthma patients [20,21]. References on inhaled corticosteroids in asthma patients are cited solely to discuss the risk of candidiasis and do not provide direct evidence for topical steroids in oncology-related OM. However, topical steroid ointments used in the context of head and neck radiation therapy have demonstrated safety and efficacy [22-25].

Among the six included studies, several reported statistically significant reductions in OM incidence and severity, with no cases of oral candidiasis or other steroid-related adverse events. These findings are particularly compelling in light of persistent concerns regarding the safety of topical steroids, concerns often extrapolated from experiences with inhaled corticosteroids. Notably, the RCTs by Niikura et al. [10] and Kuba et al. [11] demonstrated clear clinical benefit and suggested that adjunctive use of professional oral hygiene may enhance efficacy.

Despite these promising findings, the current evidence base is limited by the small number of high-quality studies, heterogeneity in steroid formulations and dosing regimens, reliance on single-institution studies, and potential publication bias. These limitations restrict the generalizability of the findings. Moreover, most studies were conducted in single institutions with relatively small sample sizes, limiting generalizability. Despite these limitations, topical corticosteroids may represent a valuable addition to supportive care protocols for patients undergoing chemotherapy or targeted therapy. Integration with professional oral care could further enhance efficacy, and accumulation of robust evidence may support guideline updates and broader clinical adoption, particularly in countries where approved formulations are lacking.

Future research should prioritize large-scale, multicenter randomized controlled trials using standardized treatment protocols to further evaluate both efficacy and long-term safety. Investigating optimal combinations, such as pairing topical steroids with antifungal prophylaxis or novel agents like honey-based therapies, may also enhance clinical outcomes. Importantly, these findings may inform revisions to existing clinical guidelines, including those from MASCC/ISOO, which currently do not recommend topical steroids for OM prevention. In Japan and other countries where regulatory and logistical barriers hinder clinical adoption, the accumulation of robust evidence could support efforts toward formal approval and broader implementation.

In summary, topical corticosteroids represent a promising yet underutilized strategy for managing OM during cancer pharmacotherapy. With further validation, they may become a valuable component of supportive oncology care, particularly in regions where alternatives are limited or cost-prohibitive.

## Conclusions

This scoping review highlights the potential of topical corticosteroid therapy, administered via mouthwash or ointment, as a safe and effective strategy for the prevention and management of OM in patients undergoing cancer chemotherapy or targeted therapy. The reviewed studies, including randomized controlled trials, consistently reported reductions in the incidence and severity of OM without significant adverse effects such as oral candidiasis. When combined with professional oral care, topical steroids may provide a valuable addition to current supportive care protocols.

Nevertheless, the current evidence base remains limited by small sample sizes, heterogeneous study designs, and a lack of standardized treatment regimens. Further high-quality, multicenter trials are needed to establish optimal formulations, dosing schedules, and long-term safety. Addressing regulatory and practical barriers, particularly in countries such as Japan, will also be essential to facilitate broader clinical adoption.
